# U.S. Adult Perspectives on Facial Images, DNA, and Other Biometrics

**DOI:** 10.1109/tts.2021.3120317

**Published:** 2021-10-18

**Authors:** Sara H. Katsanis, Peter Claes, Megan Doerr, Robert Cook-Deegan, Jessica D. Tenenbaum, Barbara J. Evans, Myoung Keun Lee, Joel Anderton, Seth M. Weinberg, Jennifer K. Wagner

**Affiliations:** Mary Ann and J. Milburn Smith Child Health Outcomes, Research, and Evaluation Center, Ann and Robert H. Lurie Children’s Hospital of Chicago, Chicago, IL 60611 USA; Department of Pediatrics, Feinberg School of Medicine, Northwestern University, Evanston, IL 60208 USA; Department of Electrical Engineering, ESAT/PSI, Medical Imaging Research Center, and Department of Human Genetics, KU Leuven, 3000 Leuven, Belgium; Sage Bionetworks, Seattle, WA 98121 USA; School for the Future of Innovation in Society, Arizona State University, Washington, DC 20006 USA; Department of Biostatistics and Bioinformatics, Duke University School of Medicine, Durham, NC 27705 USA; Levin College of Law and the Wertheim College of Engineering, University of Florida, Gainesville, FL 32611 USA; Center for Craniofacial and Dental Genetics, Department of Oral and Craniofacial Sciences, University of Pittsburgh, Pittsburgh, PA 15260 USA; Center for Craniofacial and Dental Genetics, Department of Oral and Craniofacial Sciences, University of Pittsburgh, Pittsburgh, PA 15260 USA; Center for Craniofacial and Dental Genetics, Department of Oral and Craniofacial Sciences, University of Pittsburgh, Pittsburgh, PA 15260 USA; Center for Translational Bioethics and Health Care Policy, Geisinger, Danville, PA 17822 USA. She is now with the Law, Policy, and Engineering Initiative, School of Engineering Design, Technology, and Professional Programs, Pennsylvania State University at University Park, University Park, PA 16802 USA

**Keywords:** DNA, ethics, face recognition, law, privacy, technology social factors

## Abstract

Applications of biometrics in various societal contexts have been increasing in the United States, and policy debates about potential restrictions and expansions for specific biometrics (such as facial recognition and DNA identification) have been intensifying. Empirical data about public perspectives on different types of biometrics can inform these debates. We surveyed 4048 adults to explore perspectives regarding experience and comfort with six types of biometrics; comfort providing biometrics in distinct scenarios; trust in social actors to use two types of biometrics (facial images and DNA) responsibly; acceptability of facial images in eight scenarios; and perceived effectiveness of facial images for five tasks. Respondents were generally comfortable with biometrics. Trust in social actors to use biometrics responsibly appeared to be context specific rather than dependent on biometric type. Contrary to expectations given mounting attention to dataveillance concerns, we did not find sociodemographic factors to influence perspectives on biometrics in obvious ways. These findings underscore a need for qualitative approaches to understand the contextual factors that trigger strong opinions of comfort with and acceptability of biometrics in different settings, by different actors, and for different purposes and to identify the informational needs relevant to the development of appropriate policies and oversight.

## I. Introduction

BIOMETRICS are increasingly used for many purposes in diverse societal contexts. Understanding public perspectives regarding distinct biometric modalities [1] (e.g., facial images, DNA, fingerprints, iris/retinal scans, palm scans, and voice exemplars) is important for designing human-centered practices and policies for their implementation in any context and for the responsible stewardship of biometrics and associated data. Empirical data, however, remain scant and often pertain only to one specific biometric modality [2] or one specific use case [3], [4]. Very few surveys of U.S. perspectives regarding biometrics and their diverse applications have been reported [5], [6]. These reports suggest that even when individuals are comfortable with biometrics generally, contextual factors are key and further suggest that comfort with a particular biometric modality is not necessarily consistent with either privacy considerations or concerns about the potential misuse of the biometrics and associated data. Moreover, the limited prior data available suggest sociodemographic factors might have influences on attitudes about biometrics, including comfort using biometrics in certain settings and acceptability of the use of biometrics by various entities and for various societal purposes. For example, individuals of higher socioeconomic status have been reported to be more comfortable with biometrics across multiple social contexts [6], and Black and Hispanic individuals have been reported to view some uses of biometrics more critically than White individuals [3]. Given the escalating public discourse in the United States regarding structural racism, data and concomitant power disparities, and dataveillance concerns, it is reasonable to hypothesize that age, racial and ethnic background, gender, and political leanings might influence U.S. adult perspectives on biometrics and their many possible applications in society. There remains a need to understand “how viscerally people will respond to biometrics” and sector-specific privacy concerns with biometrics [7].

## II. Survey Methods

### A. Survey Design and Administration

Survey questions were designed and programmed using Qualtrics LLC (Provo, UT, USA). Several questions were designed to enable direct comparisons to the empirical data published by others [3], [5], [6] by using similar or identical phrasing and question type. To eliminate the possibility of the content of some questions unintentionally priming or influencing responses to other questions, respondents were not able to review or change their answers to questions in one section of the survey after they progressed to a subsequent section. To minimize the likelihood that respondents would become fatigued or disinterested before completing the questions that could lead to increased item nonresponse rates and diminished data quality, the initial survey instrument was separated topically into two shorter instruments with overlapping questions: one instrument focused on biometrics in a wide variety of societal contexts, whereas the other instrument focused on biometrics in health care and research contexts (the results of which are reported elsewhere [8]). The societal contexts instrument is provided as Appendix 1 in the supplementary material, and the overlapping questions from the second survey instrument are provided as Appendix 2 in the supplementary material.

Anonymous responses to each survey from a representative sample of the online U.S. adult population were obtained using Qualtrics Panels, a panel aggregation service provided by Qualtrics that allows panel participants to earn modest incentives by completing surveys on a computer or mobile device. Eligibility criteria for these surveys required individuals to be located in the United States and at least 18 years of age.

Survey responses were collected between November 24, 2020 and December 14, 2020 with quota constraints applied to ensure the response samples would approximate the U.S. Census demographic categories for geographic region (Northeast, Midwest, West, and South), age group (18–24, 25–34, 35–44, 45–54, 55–64, and 65+), gender, and racial/ethnic background. The Qualtrics Panels project manager delivered anonymous response samples for both surveys to research personnel on December 15, 2020.

### B. Research Approvals

The University of Pittsburgh IRB determined this study (STUDY20070193) to be an exempt protocol on September 11, 2020. On October 1, 2020, the Geisinger IRB determined that the research activities (IRB#2020-0926) to be performed at Geisinger did not involve human subjects as defined in 45 CFR 46.102(f).

### C. Statistical Analysis

Responses to survey questions were either nominal or ordinal in scale and were coded for analysis. We used Pearson’s Chi-squared test to analyze survey questions with nominal responses. For questions with ordinal responses, we used the Wilcoxon sign rank test to assess whether the underlying distribution of responses deviated significantly from the sample median response. In addition, Kruskal—Wallis tests were used to assess the effect of demographic variables (age, sex, race, etc.) on our outcomes; this was followed by *post-hoc* Dunn tests with Bonferroni adjustment for multiple comparisons. We used ordinal polychoric correlations to investigate the relationship between prior experience and comfort with biometrics. Our nominal threshold for statistical significance was set at 0.05. We used R to perform all analysis [9]. Statistical test results are provided in Appendix 3 in the supplementary material.

## III. Survey Results

### A. Demographics of Survey Respondents

A total of 4048 survey responses were obtained and analyzed, involving 2038 respondents to the societal contexts survey instrument and 2010 respondents to the overlapping questions contained in the separate survey instrument. Table I displays the self-reported age, geographic region, gender, racial and ethnic background, educational attainment, household income, and general political views of the respondents for the overall response sample and the societal context survey sample. We have made the survey data available via Synapse [10].

### B. Comfort With Biometrics Generally

**Fig. 1.**
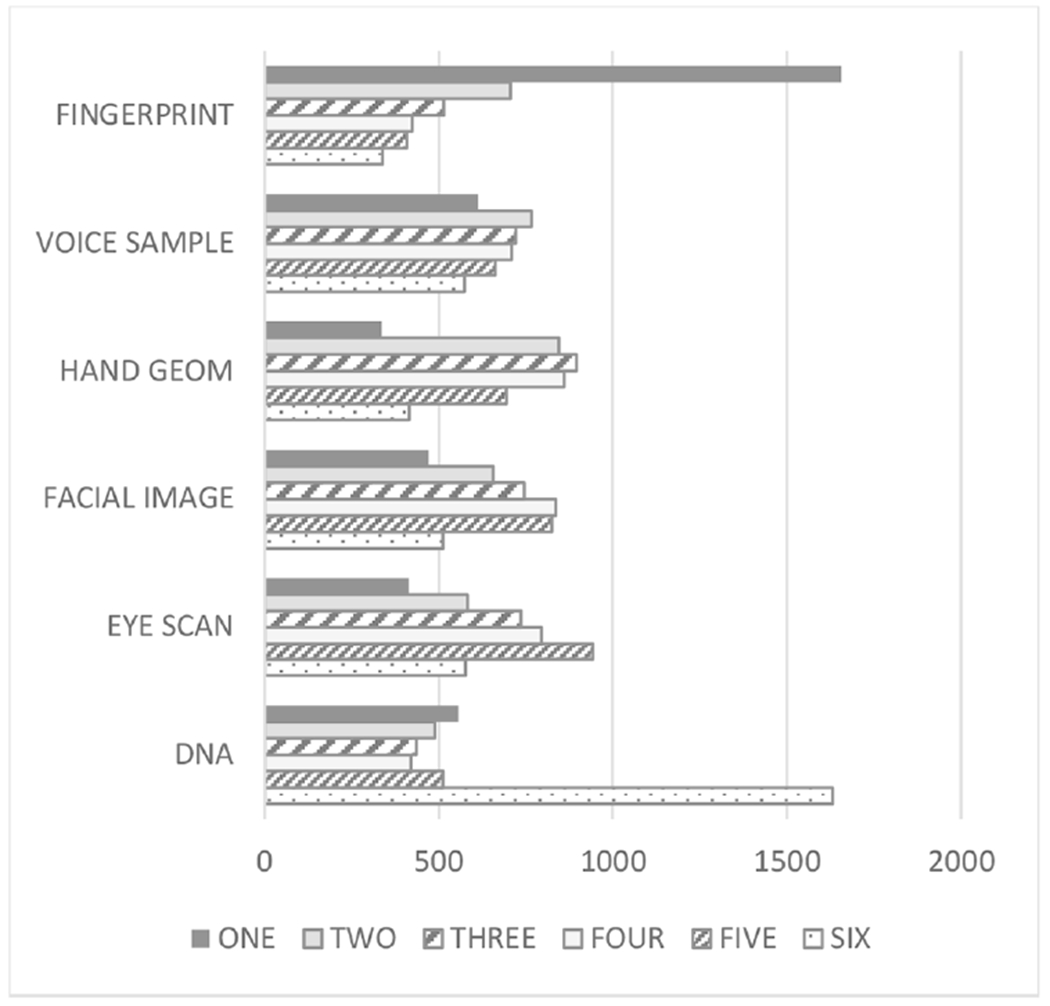
U.S. adults’ ranking of comfort with six types of biometrics. One is the most comfortable ranking, and six is the least comfortable ranking.

Survey participants ranked six types of biometrics (fingerprint, voice sample, facial image, eye scan, hand geometry, and DNA sample) in order of which they were most comfortable providing to an organization for any purpose. As shown in Fig. 1, of the 4048 respondents, 40.9% (1656/4048) ranked fingerprints as the biometric with which they were most comfortable providing to an organization for any purpose, and 40.3% (1632/4048) ranked DNA as the biometric with which they were least comfortable. Interestingly, 38.4% (1556/4048) of respondents indicated they were more comfortable with DNA than they were with facial imaging.

**Fig. 2.**
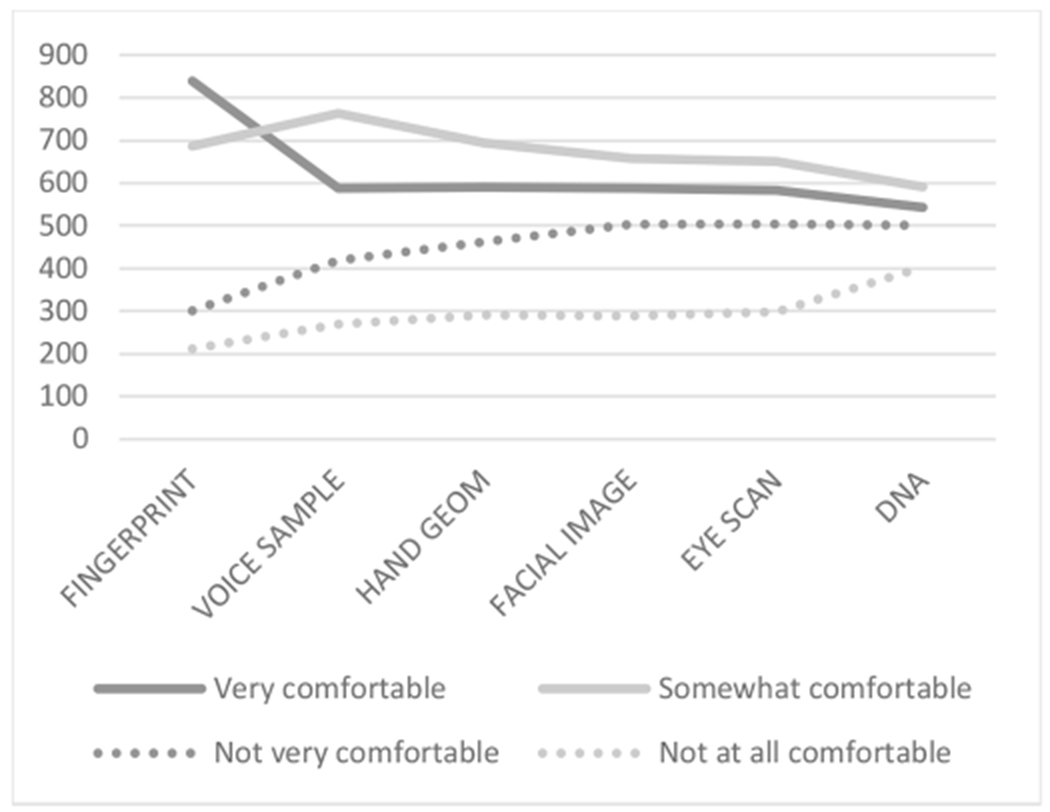
U.S. adults’ comfort with six types of biometrics. The biometric types are shown in order of highest observed expressions of comfort (fingerprints) to lowest (DNA).

Among those survey participants (*N*=2038) asked to indicate their general level of comfort (i.e., very comfortable, somewhat comfortable, not very comfortable, or not at all comfortable) providing biometric information to an organization for any purpose, we found an expected correlation between reported prior experience and level of comfort with that specific biometric, with observed correlation values ranging from 0.285 (hand geometry) to 0.418 (fingerprint) and all at *p*-values <0.001. As shown in Fig. 2—and similar to findings reported previously [5]—respondents overall were generally more comfortable than not with each of the biometric types, with the highest levels of comfort being expressed for fingerprints (74.8%, 1525/2038 reportedly very or somewhat comfortable) and the lowest levels of comfort expressed for DNA samples (55.6%, 1134/2038 reportedly very or somewhat comfortable). Levels of comfort expressed for voice samples, hand geometry, facial imaging, and eye scans were 66.2% (1350/2038), 63.0% (1284/2038), 61.1% (1246/2038), and 60.6% (1235/2038), respectively. Demographics had small or no effects on reported levels of comfort with the six different biometrics.

Respondents who indicated they were not very comfortable or not at all comfortable with any specific biometric (60.6%, 1235/2038) were asked to identify from a list of options the single main reason behind that discomfort. Nearly one-third (28.9%, 353/1235) indicated it was an invasion of personal privacy; 22.9% (283/1235) indicated that the purpose for collecting or using the biometric information is important for them to know; 18.4% (227/1235) pointed to concerns about identity theft; 10.9% (134/1235) expressed worry about government surveillance; and 8.9% (110/1235) indicated the policy for keeping or destroying the biometric information is important for them to know; and only 2.2% (27/1235) expressed worry about targeted marketing by advertisers as the main source of their discomfort. The remainder (8.2%, 101/1235) indicated the main reason for their discomfort was something else. Despite our study offering two reasons not previously explored, the most cited reason (that this is an invasion of privacy) was consistent with results reported elsewhere [5].

In addition to general comfort with biometrics, half of the survey participants (*N* = 2038) were asked to indicate how comfortable they were with a specific biometric modality in a specific social context using a scale from 1 (very uncomfortable) to 6 (very comfortable). A majority of respondents expressed comfort with either fingerprints (67.1%, 1367/2038 indicating 4, 5, or 6) or facial recognition technology (FRT) (59.3%, 1208/2038 indicating 4, 5, or 6) to unlock smartphones instead of a passcode. Two scenarios addressed biometrics to access financial information: respondents were divided as to their comfort with the use of voiceprints to verify the identity of a credit card customer when seeking information about their credit card account over the phone (49.8%, 1014/2038 indicating 4, 5, or 6), but roughly three-fifths of respondents were comfortable with a fingerprint to access bank account information via a smartphone app (60.4%, 1237/2038). Of the seven scenarios involving FRT, a majority of respondents indicated they were not comfortable (indicating a 1, 2, or 3) with the technology in four scenarios: 1) retailers using it to track store movements to later target customers with advertising (63.2%, 1287/2038); 2) a people search company (i.e., a company offering products to enable individuals to search for and reconnect with people they know such as classmates, coworkers, neighbors, and friends [11]) using it to link profiles of people across social media sites (62.3%, 1270/2038); 3) a homeowner’s association using it to track the movements of people on its streets and sidewalks (63.3%, 1289/2038); and 4) a coffee shop using it rather than ID cards to administer its customer loyal program (60.7%, 1237/2038). Whereas respondents were generally uncomfortable with FRT being used by a homeowner’s association to track community movements as previously mentioned, respondents were generally comfortable with smart doorbells incorporating FRT to notify a homeowner when particular people approach a front door (with 58.5%, 1192/2038 indicating a 4, 5, or 6). Additionally, whereas most respondents were uncomfortable with retailers using facial recognition technologies for targeted advertising, a majority were comfortable with retailers using it to detect when people who have been banned from the stores (such as shoplifters) have entered the establishment (54.8%, 1117/2038 indicating a 4, 5, or 6). A majority of respondents were comfortable with employers using fingerprint scans in lieu of timecards to track when employees check in and out of work (60.3%, 1228/2038 indicating 4, 5, or 6) as well as a gym allowing members to check-in with a fingerprint scan rather than a membership ID card (55.6%, 1134/2038 indicating a 4, 5, or 6). Demographics had small or no effects on reported levels of comfort with biometrics in these scenarios.

### C. Perceived Trust in Diverse Social Actors to Use Specific Biometrics Responsibly

**Fig. 3.**
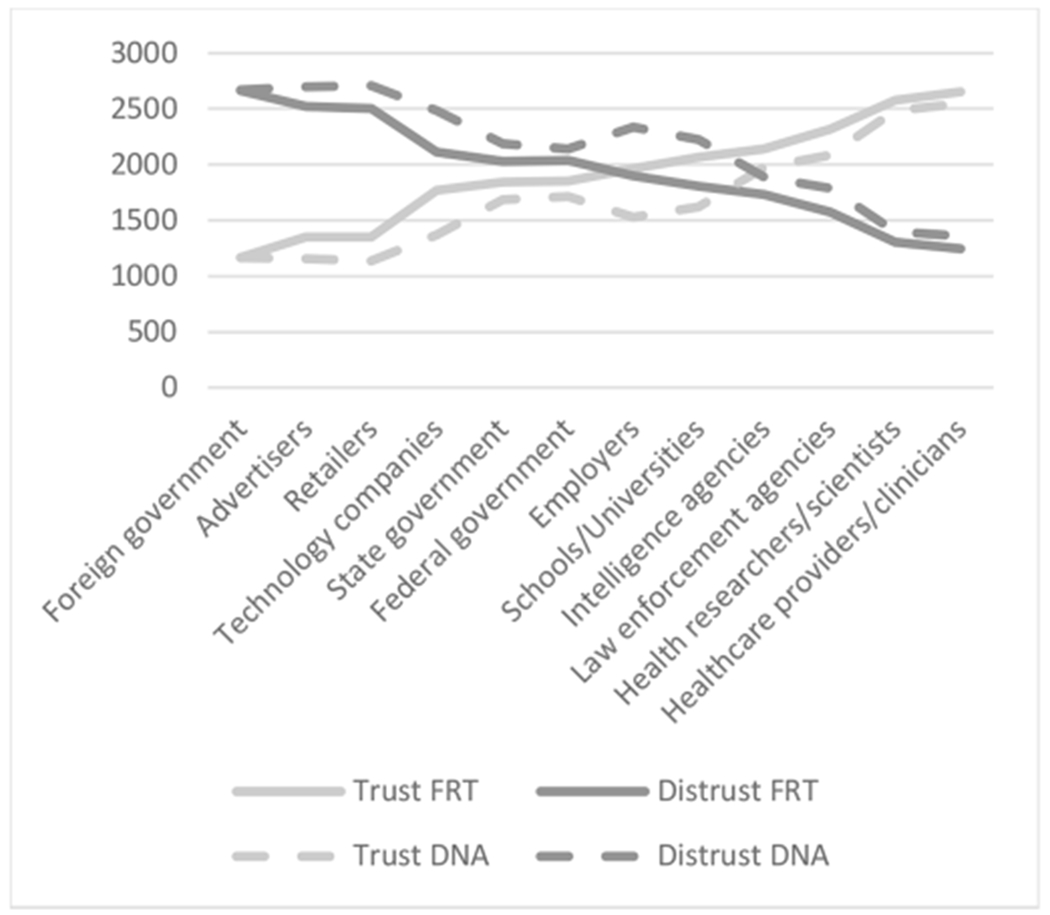
U.S. adults’ trust in social actors to use of two types of biometrics, facial imaging/FRT and DNA, and associated data responsibly. Perceptions of responsible use were similar for both facial imaging and DNA. Order from left to right is by least trust in FRT to most.

Survey participants (*N* = 4048) were asked to report how much they trust certain social actors to use either facial images and facial imaging data or DNA and DNA data responsibly. Social actors included in the query were advertisers, technology companies, law enforcement agencies, intelligence agencies, health researchers and scientists, healthcare providers and clinicians, employers, schools and universities, retailers, and state, federal, and foreign governments. Fig. 3 displays the similar trends observed in respondents’ perceptions of trustworthiness of social actors and both biometric types. A majority of respondents trusted law enforcement agencies, intelligence agencies, health researchers/scientists, and healthcare providers/clinicians to use both types of biometrics responsibly, and a majority of respondents indicated advertisers, tech companies, retailers, and state, federal, and foreign governments were not trusted to handle either type of biometric responsibly. A divergence in biometric acceptability was found regarding trust in employers and schools/universities, with most respondents indicating those social actors could be trusted with facial imaging and imaging data but not DNA or DNA data. The respondents’ highest levels of trust related to health researchers/scientists (66.4%, 2576/3881 and 64.0%, 2489/3890 reporting somewhat or a great deal of trust for use of facial imaging and DNA, respectively) and healthcare providers/clinicians (68.1%, 2654/3900 and 65.2%, 2548/3908 reporting somewhat or a great deal of trust for use of facial imaging and DNA, respectively). Denominators vary due to item nonresponse.

Demographics had small or no effects on reported levels of trust that social actors would use facial images and facial imaging data responsibly or DNA and DNA data responsibly except for age being found to have a moderate effect on trust in advertisers’ and foreign governments’ use of either type of biometric and biometric data and also age having a moderate effect on trust in retailers’ use of DNA and DNA data.

### D. Acceptability of Specific Biometric in Societal Contexts

**Fig. 4.**
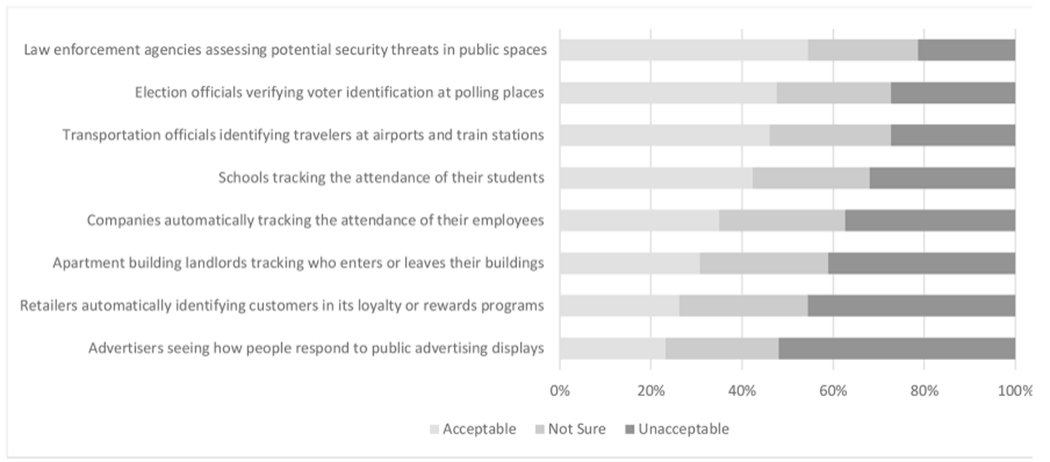
Acceptability of a specific biometric in eight scenarios. Six of the eight scenarios failed to elicit a majority reaction either of acceptability or unacceptability, suggesting nuances are important.

Some survey participants (*N*=2038) were given a series of eight scenarios and asked to indicate whether such use of one specific biometric modality (FRT) was acceptable or unacceptable in that context. Four of the scenarios were identical to those asked previously by the Pew Research Center [3]. As shown in Fig. 4, there was only one scenario for which most respondents (54.4%, 1061/1950) indicated FRT was acceptable: law enforcement agencies assessing potential security threats in public spaces. Likewise, there was only one scenario for which most respondents indicated the use of FRT was unacceptable: advertisers seeing how people respond to public advertising displays (52.0%, 1001/1926). No other scenario elicited a majority of respondents expressing that the use was either acceptable or unacceptable. For each of the eight scenarios, approximately one-quarter of respondents indicated they were not sure. Approximately 9.1% (185/2038) indicated all eight uses were acceptable, whereas 5.9% (121/2038) indicated all eight uses were unacceptable. Demographics had small or no effects on the acceptability of FRT in the scenarios posed.

### E. Perceived Effectiveness of Specific Biometric

Survey participants (*N* = 4048) indicated how effective they thought one specific biometric modality (FRT) was at five distinct tasks: 1) identifying individuals; 2) assessing someone’s sex or gender; 3) assessing someone’s race or ethnicity; 4) detecting someone’s emotions or feelings; and 5) diagnosing someone’s medical conditions. FRT was generally perceived to be effective at all tasks. More than 82% of respondents (3119/3794) indicated FRT is somewhat or very effective at accurately identifying people. A majority of respondents indicated FRT is similarly effective in assessing sex or gender and assessing race or ethnicity (64.6%, 2389/3697 and 65.2%, 2431/3728, respectively). Respondents were more evenly divided on perceived effectiveness of facial recognition in detecting emotions or feelings and diagnosing medical conditions, with 50.3%, 1848/3676 and 49.6%, 1804/3639 indicating FRT is somewhat or very effective, respectively. Demographics had small or no effects on perceived effectiveness ratings.

## IV. Discussion

This study of public perspectives on biometrics in the United States offers a glimpse at factors relevant for understanding how the use of biometrics is or could be supported or opposed in a variety of societal contexts. Yet the findings are somewhat unremarkable, their significance mainly being in what they are unable to tell us. Accordingly, they must be viewed as an important foundational step but not a definitive word on public perspectives of biometrics and their many uses.

Whereas we observed a correlation between experience and comfort with the six types of biometrics, it is not possible to decipher whether comfort is due to prior experience or, conversely, whether the prior experience is due to being more comfortable with the biometric and transfer of biometric data to an organization. Additionally, there is a possibility that respondents were thinking about the process or means with which a biometric would be obtained or measured when ranking biometrics or indicating their level of comfort with a specific biometric rather than weighing data risks. For example, when ranking comfort with facial imaging above eye (iris/retinal) scans, it would be important to know to what extent respondents were thinking about the ease and non-invasive nature of capturing a photograph versus uncertain physical discomforts from an eye scan process with which they are unfamiliar or, alternatively, whether respondents’ rankings were influenced by the ways in which facial images might be considered publicly available and accessible to laypersons rather than iris scans being less accessible due to technical expertise to capture and decipher them.

Our examination of facial imaging and DNA specifically revealed that general trends regarding perceived trust in social actors to use those two biometrics and related data responsibly hold true regardless of which biometric is contemplated. That law enforcement and intelligence agencies are trusted by a majority but state and federal governments are not trusted suggest that purpose creep is a major concern (as also reported by [6]), as law enforcement and intelligence agencies are, in fact, part of those governmental entities. To our knowledge, no data are yet available to gauge 1) public awareness of facial recognition and oppressive dataveillance of Uighurs in China [12] or of the U.S. Department of Homeland Security’s proposed expansion of biometrics for dataveillance of individuals within the United States [13] or 2) possible failures to imagine risks and harms when the public is asked to consider whether specific social actors can be trusted to be responsible with facial imaging, DNA, and other biometrics and related data. Moreover, it is notable that our data were collected several weeks prior to the violent attack at the U.S. Capitol on January 6, 2021 [14], an event prompting both extensive uses of FRT by law enforcement to identify perpetrators [15] and renewed calls for bans on law enforcement use of facial recognition technology [16]. As legislation involving facial recognition and other biometrics is considered, it would be important for policymakers and academics to seek clarity through deliberative democracy research approaches [17].

Trust in health researchers and healthcare providers using biometrics is worth further examination. For example, further research is necessary to understand healthcare applications for biometrics (e.g., [4], [8], [18], and [19]) and how individuals view partnerships between healthcare providing organizations and technology companies for the implementation of biometric programs such as FRTs given the observed high levels of trust in health professionals (whether care providers or researchers) and low levels of trust in technology companies to use such biometrics tools responsibly.

The acceptability of eight distinct scenarios using FRT as we studied them failed to elicit a majority viewpoint on six of the eight scenarios, which underscores the importance of nuance and particulars to—i.e., informational needs of—individuals before they are moved by logic or emotion to respond in a decisive way about the acceptable uses of biometrics. For example, the scenarios we presented did not include specific details regarding named social actors, biometric collection procedures, data retention and destruction policies, or costs, benefits, and viable alternatives to be considered. That a sizable proportion of the respondents to our surveys occupied an ambiguous or ambivalent position on the acceptability of FRT (despite growing debates on point [20], [21]) might dampen public pressure for sound policies. This ambiguous or ambivalent position could suggest individuals feel helpless (e.g., perceive the uses of biometrics or policies governing them as out of their control) or perhaps simply that more zealous opinions are dependent on very specific contextual details. Public perspectives change over time as people learn about real-world uses of biometrics and controversies surrounding them. Paradoxically, practices are more easily changed before they become entrenched. It is imperative that we seek a better understanding of individuals’ informational needs and facilitate opportunities for individuals to develop informed opinions on these issues before, in the absence of a collective will, policymakers—or, more likely, corporations—make normative decisions that will be difficult to change or reverse once established. Particularly in light of the U.S. Government Accountability Office’s report that at least ten federal agencies plan to expand their use of FRTs through fiscal year 2023 [22], it is both urgent and important that U.S. public perspectives be examined more closely so that they can inform governmental policy.

We expected to find stark divergences of opinions among historically marginalized groups based on mounting attention given to dataveillance issues, but sociodemographic factors did not strongly influence perspectives on biometrics in any obvious way. Contrary to prior reports [3], racial and ethnic background had only small effects on perspectives regarding biometrics. For example, despite documented biases in the poor performance of FRT in identifying individuals who are not White men [23]–[25], racial and ethnic background had no significant effect on the perceived effectiveness of FRT in assessing race or ethnicity. Age was the only variable to show moderate effects and only with regard to perceived trust in specific social actors (advertisers, retailers, and foreign governments) to use facial imaging and DNA responsibly, with levels of trust being lower among older respondents. This result could suggest levels of trust in these social actors’ use of biometrics will increase over time as younger individuals age or, conversely, levels of trust will wane with the accumulation of life experience.

Compiled online research panels (like Qualtrics Panels) have been demonstrated to be reliable and effective strategies for nationally representative response data [26]–[28], and researchers have shown Qualtrics Panels perform relatively well in comparison with more arduous and expensive probabilistic sampling approaches [29]. However, there are potential limitations to our study attributable to our use of Qualtrics Panels. Individuals who opt-in to such panels to participate in surveys might be less concerned about data privacy and have a less critical view of biometrics than nonparticipants. Additionally, the mean time for completion of each of the surveys we administered was approximately 10 min (9.68 and 10.35 min, respectively), suggesting that our results reflect surface, visceral feedback prone to influence by habitual interactions with biometrics (such as unlocking a smartphone with a thumbprint). Understanding deeper perspectives of engaged individuals who are more thoroughly contemplating the issues and their broader implications is needed. Whereas quantitative data such as those reported here are valuable for exploring public perspectives, the results underscore the need for a qualitative approach that delves more deeply into the contextual factors underlying and shaping these perspectives.

## Supplementary Material

supp1-3120317

supp2-3120317

supp3-3120317

## Figures and Tables

**TABLE I:** Demographics of Survey Respondents

Demographic		All Respondents		Respondents to Societal Contexts Survey	
		N = 4048	%	N = 2038	%
Age	18-25	524	12.9	263	12.9
	26-35	728	18.0	364	17.9
	36-45	678	16.7	339	16.6
	46-55	671	16.6	352	17.3
	56-65	675	16.7	337	16.5
	66-75	640	15.8	326	16.0
	76+	132	3.3	57	2.8

Geographic Region	South	1491	36.8	729	35.8%
	West	999	24.7	520	25.5%
	Midwest	840	20.8	441	21.6%
	Northeast	713	17.6	345	16.9%

Gender	Woman	2029	50.1	1039	51.0
	Man	1943	48.0	965	47.4
	Non-binary	21	0.52	9	0.44
	Transgender (including Man, Transgender; Woman, Transgender; or Transgender)	13	0.32	5	0.25

Racial and Ethnic Background	American Indian or Alaska Native	56	1.38	17	0.83
	Asian	172	4.2	92	4.5
	Black, African American, or African	473	11.7	238	11.7
	Hispanic, Latino, or Spanish	470	11.6	235	11.5
	Native Hawaiian or other Pacific Islander	21	0.52	3	0.15
	White	2466	60.9	1282	62.9
	Black, African American, or African & Hispanic, Latino, or Spanish	9	0.22	5	0.25
	Black, African American, or African & White	52	1.28	22	1.08
	Hispanic, Latino, or Spanish & White	123	3.04	62	3.04
	Other combination of two or more categories	129	3.19	55	2.70

Educational Attainment	Grade 11 or below	218	5.4	113	5.5
	Grade 12 or GED	912	22.5	472	23.2
	1 to 3 years after high school	1221	30.2	640	31.4
	College 4 years or more	967	23.9	468	23.0
	Advanced degree	688	17.0	328	16.1

Household Income	Less than $25,000	823	20.3	423	20.8
	$25,000 - $49,999	1058	26.1	577	28.3
	$50,000 - $74,999	680	16.8	347	17.0
	$75,000 - $99,999	496	12.3	228	11.2
	$100,000 - $149,999	453	11.2	222	10.9
	$150,000 or more	368	9.1	160	7.9

Political Views	Conservative	1099	27.1	542	26.6
	Moderate	1568	38.7	818	40.1
	Liberal	1017	25.1	506	24.8

Totals for each demographic item do not necessarily sum to N=4048 for all respondents or N=2038 for societal survey respondents due to item nonresponse or selections (such as “I prefer not to answer.”) that are not displayed.

## Appendix

Appendix 1 in the supplementary material contains the questions included in the biometrics in the social contexts survey instrument. Appendix 2 in the supplementary material contains the overlapping questions administered in the second survey instrument. Appendix 3 in the supplementary material contains the statistical test results.
