# Supplementary material for: U.S. Adult Perspectives on Facial Images, DNA, and Other Biometrics
Source: IEEE Trans Technol Soc. Author manuscript; Available in PMC 2022 Mar 30. (PMC8965792; doi:10.1109/tts.2021.3120317)
Supplement: supp2-3120317 [file NIHMS1788965-supplement-supp2-3120317.pdf]

APPENDIX 2. OVERLAPPING, IDENTICAL QUESTIONS ON THE HEALTH CARE AND RESEARCH CONTEXTS SURVEY

Notes about Question Design:
Questions 1-6 were modeled on those used by the *All of Us*[SM] Research Program (available at https://www.researchallofus.org/survey-explorer/) but were streamlined for quicker administration. Questions 7 and 8 were adapted from those used by [3] and reported by [5]. Question 11 was modeled on survey results reported by [5] to omit typing dynamics and signature dynamics and to include DNA. Question 14 was modeled on survey results reported by [5] to include DNA. Question 19 was adapted from [3]. Question 20 was adapted from [3] to explore trust in additional social actors beyond response items (a)-(c), and Q21 was modeled to explore parallel perspectives for DNA. Question 22 was adapted from [3] to explore acceptability of facial recognition technologies in different contexts as items (h)–(o). Question 23 was adapted from [3] to explore perceived effectiveness in two additional tasks displayed as response items (d) and (e).

<u>Basic Questions About You</u>

1. In which age group do you belong?
    a. 18 to 25 years old
    b. 26 to 35 years old
    c. 36 to 45 years old
    d. 46 to 55 years old
    e. 56 to 65 years old
    f. 66 to 75 years old
    g. 76 years and older
2. Where in the United States do you currently live? [pull-down menu for 50 states, district, and 5 US territories]
3. Which categories describe you? Select all that apply. Note, you may select more than one group.
    a. American Indian or Alaska Native
    b. Asian
    c. Black, African American, or African
    d. Hispanic, Latino, or Spanish
    e. Middle Eastern or North African
    f. Native Hawaiian or other Pacific Islander
    g. White
    h. None of these fully describe me. I identify as: (free text)
    i. I prefer not to answer.
4. What terms best express how you describe your gender identity? Select all that apply.
    a. Man
    b. Woman
    c. Non-binary
    d. Transgender
    e. None of these describe me. I identify as: (free text)
    f. I prefer not to answer.
5. What is the highest grade or year of school you completed?
    a. Never attended school or only attended kindergarten
    b. Grades 1 through 4 (Primary school)
    c. Grades 5 through 8 (Middle school)
    d. Grades 9 through 11 (Some high school)
    e. Grade 12 or GED (High school graduate)
    f. 1 to 3 years after high school (Some college, Associate’s degree, or technical school)
    g. College 4 years or more (College graduate)
    h. Advanced degree (Master’s, Doctorate, etc.)
    i. I prefer not to answer.
6. What is your annual household income from all sources?
    a. Less than $25,000
    b. $25,000 - $49,999
    c. $50,000 - $74,999
    d. $75,000 - $99,999
    e. $100,000 - $149,999
    f. $150,000 or more
    g. I prefer not to answer.
7. Which most closely describes your political views today?
    a. Democrat
    b. Independent but leaning Democrat
    c. Independent but leaning Republican

    d. Republican
    e. None of these fully describe me.
    f. I prefer not to answer.
8. Which most closely describes your political views in general today?
    a. Liberal
    b. Moderate
    c. Conservative
    d. I prefer not to answer.

<u>Your Relevant Experiences</u>

9. Have you ever provided a DNA sample for any reason?
    a. Yes
    b. No
    c. Not sure
10. Have you ever had medical imaging of your head or face—such as an MRI scan (magnetic resonance imaging scan), CT scan (computed tomography scan), or medical photographs of your head or face?
    a. Yes
    b. No
    c. Not sure
11. Indicate your experience with each of these six types of biometrics. For example, perhaps you have used biometrics (a fingerprint or face scan) to unlock your smartphone or computer; perhaps you used biometrics (such as a palm scan) to enter a building with restricted access. Please think about all possible contexts.

Yes, I have experienced this type of biometric.	No, I have not experienced this type of biometric.	I am not sure if I have experienced this type of biometric.

    a. Fingerprint (digital or ink)
    b. Voice sample
    c. Facial image
    d. Eye (iris/retina) scan
    e. Hand geometry (palm) scan
    f. DNA (genetic profile or fingerprint)

<u>Your Perspectives About Biometrics</u>

12. [Question Omitted from this Survey]
13. [Question Omitted from this Survey]
14. Rank the types of biometrics in the order from the type you are most comfortable providing (1) to the type you are least comfortable providing (6) to an organization. [options randomized]
    a. Fingerprint
    b. Facial image
    c. Voice sample
    d. Eye (iris/retinal) scan
    e. Hand geometry (palm) scan
    f. DNA (genetic profile or fingerprint)
15. How does your opinion of biometrics today compare with your opinion of biometrics five years ago?
    a. My opinion of biometrics is **MORE** favorable today than it was five years ago.
    b. My opinion of biometrics has **NOT CHANGED** over the past five years.
    c. My opinion of biometrics is **LESS** favorable than it was five years ago.
16. Which statement best describes how the COVID-19 pandemic has affected your opinion of the use of biometrics in society?
    a. The pandemic has made me **MORE** comfortable with use of biometrics in society.
    b. The pandemic has **NOT CHANGED** my opinion regarding the use of biometrics in society.
    c. The pandemic has made me **LESS** comfortable with use of biometrics in society.
17. [Question Omitted from this Survey]

Your Perspectives on Related Issues

18. How concerned are you about the potential misuses of your personal information in general?
    a. Very concerned
    b. Somewhat concerned
    c. Not very concerned
    d. Not concerned at all
    e. Don’t know

19. How much, if at all, do you trust the following groups to use **facial recognition technology** responsibly?

A great deal	Somewhat	Not too much	Not at all	No answer

a. Advertisers
b. Technology companies
c. Law enforcement agencies
d. Intelligence agencies
e. Health researchers/scientists
f. Healthcare providers/clinicians
g. Employers
h. Schools/Universities
i. Retailers
j. State Government
k. Federal Government
l. Foreign Government

20. How much, if at all, do you trust the following groups to use **DNA and DNA data** responsibly?

A great deal	Somewhat	Not too much	Not at all	No answer

a. Advertisers
b. Technology companies
c. Law enforcement agencies
d. Intelligence agencies
e. Health researchers/scientists
f. Healthcare providers/clinicians
g. Employers
h. Schools/Universities
i. Retailers
j. State Government
k. Federal Government
l. Foreign Government

21. In your opinion, are biometric data protection and privacy laws adequate in the United States?
a. Yes
b. No
c. Not sure

22. In your opinion, is it acceptable or unacceptable to use facial recognition technology in the following situations? [options randomized]

Acceptable	Unacceptable	Not Sure	No Answer

a. [Item omitted from this survey]
b. [Item omitted from this survey]
c. [Item omitted from this survey]
d. [Item omitted from this survey]
e. [Item omitted from this survey]
f. [Item omitted from this survey]
g. [Item omitted from this survey]
h. Hospitals checking surgical patients' identities to avoid medical errors.
i. Hospitals tracking who enters or leaves their buildings to assess potential security threats.
j. Hospitals identifying patients who are nonresponsive, unaccompanied, or otherwise without identification to enable continuity of care.
k. Healthcare providers trying to diagnose certain conditions and diseases earlier, faster, or better.
l. Healthcare providers monitoring patient's emotions or symptoms (such as pain, fear, relief, anger, mistrust, sadness, happiness, or satisfaction).
m. Pharmacies assessing threats of insurance fraud or identity theft when prescriptions are filled.
n. Hospitals or clinics verifying staff identities for access to electronic health records, prescriptions, or building access.
o. Scientists linking diverse data sources to conduct health research.

Your Understanding of Facial Imaging Technology

23. Based on what you know, how effective do you think facial recognition technology is at the following tasks?

Very effective	Somewhat effective	Not too effective	Not effective at all	No answer

a. Accurately identifying individual people
b. Accurately assessing someone’s sex or gender
c. Accurately assessing someone’s race or ethnicity
d. Accurately detecting someone’s emotions or feelings
e. Accurately diagnosing someone’s medical conditions

[New Questions 24-27 omitted from Societal Contexts Survey]
