# Supplementary material for: U.S. Adult Perspectives on Facial Images, DNA, and Other Biometrics
Source: IEEE Trans Technol Soc. Author manuscript; Available in PMC 2022 Mar 30. (PMC8965792; doi:10.1109/tts.2021.3120317)
Supplement: supp3-3120317 [file NIHMS1788965-supplement-supp3-3120317.pdf]

Appendix 3. Statistical Test Results

Table I: Ranking of different biometrics in terms of comfort								
			Fingerprint	Voice sample	Facial image	Eye scan	Hand scan	DNA sample
Sample count	Comfort level	Coding	N					
	6 (least)	0	338	574	512	576	416	1632
	5	1	410	664	824	943	695	512
	4	2	424	711	836	795	861	421
	3	3	514	721	746	736	894	437
	2	4	706	765	658	584	846	489
	1 (most)	5	1656	613	472	414	336	557
		total	4048	4048	4048	4048	4048	4048
Wilcoxon sign rank test		Test statistic	6351136	4276966	3797990	3375205	4155888	2497620
		p-value	1.15E-209	0.0144	4.27E-05	5.82E-23	0.425	1.67E-107
		Effect size	0.486	0.038	0.064	0.155	0.013	0.346
		Magnitude	moderate	small	small	small	NS	moderate
		Sign	+	+	-	-	+	-
Kruskal–Wallis test		Covariate	Effect size					
		Age	small	NS	NS	NS	NS	small
		Region	small	NS	NS	NS	NS	small
		Race	small	small	NS	small	small	small
		Sex/Gender	NS	NS	NS	NS	NS	NS
		Education	small	NS	small	NS	NS	small
		Income	small	NS	NS	NS	NS	small
		Political philosophy	NS	NS	NS	NS	NS	NS
		DNA experience	small	small	NS	NS	NS	small
		Imaging experience	NS	NS	NS	NS	NS	small

NS = Not statistically significant.

Effect size *r* for Wilcox sign rank test: small = 0.10 < 0.29; Moderate = 0.30 < 0.49; Large = > 0.49

Effect size e^2 for Kruskal–Wallis test: Small = 0.01 < 0.079; Moderate = 0.08 < 0.259; Large = > 0.259

Sign for Wicoxon Test: + indicates a bias in the distribution toward more comfort; - indicates a bias in the distribution toward less comfort

Table II: Relationship between prior experience and comfort with biometrics

		Prior experience providing					
		Fingerprint	Voice sample	Facial Image	Eye scan	Hand scan	DNA sample
Comfort providing	Fingerprint	**0.418**	0.209	0.273	0.224	0.239	0.216
	Voice sample	0.254	**0.359**	0.296	0.202	0.245	0.171
	Facial image	0.254	0.240	**0.361**	0.243	0.279	0.190
	Eye scan	0.172	0.216	0.286	**0.329**	0.215	0.185
	Hand scan	0.259	0.205	0.268	0.221	**0.285**	0.189
	DNA sample	0.191	0.149	0.206	0.216	0.203	**0.350**

Computed as ordinal polychoric correlations; a positive correlation indicates that the more prior experience one has with biometrics the more comfort one has with providing biometrics to an organization

Bold face type indicates the strongest correlation in each column

All correlations were positive and all statistically significant at $p < 0.001$

Table III: Participants' level of comfort providing biometric data

			Fingerprint	Voice sample	Facial image	Eye scan	Hand scan	DNA sample
Sample count	Comfort level	Coding	N					
	Not at all comfortable	0	212	270	289	299	291	403
	Not very comfortable	1	301	418	503	504	463	501
	Somewhat comfortable	2	686	763	657	651	694	591
	Very comfortable	3	839	587	589	584	590	543
		total	2038	2038	2038	2038	2038	2038
Wilcoxon sign rank test		Test statistic	1608291	1396003	1323494	1308926	1344646	1173048
		p-value	1.42E-108	4.97E-44	1.49E-28	7.28E-26	1.08E-32	1.81E-07
		Effect size	0.490	0.308	0.246	0.233	0.264	0.116
		Magnitude	moderate	moderate	small	small	small	small
		Sign	+	+	+	+	+	+
Kruskal–Wallis test		Covariate	Effect size					
		Age	small	small	small	NS	NS	small
		Region	small	NS	NS	NS	small	NS
		Race	NS	NS	NS	NS	small	small
		Sex/Gender	small	small	small	small	small	small
		Education	small	small	small	small	small	small
		Income	small	small	small	small	small	small
		Political philosophy	small	NS	NS	NS	small	NS
		DNA experience	small	small	small	small	small	small
		Imaging experience	small	small	small	small	small	small

NS = Not statistically significant.

Effect size *r* for Wilcox sign rank test: small = 0.10 < 0.29; Moderate = 0.30 < 0.49; Large = > 0.49

Effect size e^2 for Kruskal–Wallis test: Small = 0.01 < 0.079; Moderate = 0.08 < 0.259; Large = > 0.259

Sign for Wicoxon Test: + indicates a bias in the distribution toward more comfort; - indicates a bias in the distribution toward less comfort

Table IV: Participants' main reason for discomfort related to providing biometric data

Reason	Count
Invasion of personal privacy.	353 (28.6%)
The reason or purpose for collecting or using biometric information is important for me to know.	283 (22.9%)
Identity theft concerns.	227 (18.4%)
Worried about government tracking or surveillance.	134 (10.9%)
The policy for keeping or destroying the biometric information is important for me to know.	110 (8.9%)
None of these. My main reason for not feeling comfortable is something else.	101 (8.2%)
Worried about targeted marketing by advertisers.	27 (2.2%)
Total	1235
Missing	803

Pearson's Chi-squared test	Covariate	Chi-squared	df	p-value
	Age	81.293	36	2.363e-05
	Region	24.238	24	NS
	Race	27.152	30	NS
	Sex/Gender	38.32	12	0.0001361
	Education	86.226	24	6.018e-09
	Income	52.735	30	0.006339
	Political philosophy	22.241	12	0.03491
	DNA experience	22.199	12	0.03535
	Imaging experience	25.335	12	0.01331

NS = Not statistically significant.

Table V: Participants' comfort with biometric data in specific societal contexts

			A bank uses a customer's fingerprint rather than a password to access the bank's smartphone app	A credit card company using a voiceprint to confirm the identity of a customer when they call about their account	A “smart” doorbell that uses facial recognition to notify a homeowner when particular people approach their front door	A smartphone using facial recognition rather than passcodes to unlock	A smartphone using fingerprints rather than passcodes to unlock	A retail store using facial recognition to detect when people who have been banned from their stores - for example, people caught shoplifting - have entered	A retail store using facial recognition to track where individual customers go in their stores and what items those customers look at so they can later send those customers targeted advertisements	A people search company using facial recognition to link the profiles of people across different social media sites	A homeowner's association using facial recognition to track the movements of people on its streets and sidewalks	An employer using fingerprint scans rather than timecards for people to check in at work	A coffee shop using facial recognition rather than id cards to administer their customer loyalty program, with cameras identifying people as they approach the counter	A gym having their members check-in using a fingerprint scan rather than an id card
Sample count	Comfort level	Coding	N											
	1 (least)	0	324	360	265	273	212	318	651	623	717	271	568	342
	2	1	162	282	208	177	173	224	283	292	272	196	297	214
	3	2	315	382	373	380	286	379	353	355	300	343	372	348
	4	3	390	403	410	387	398	379	304	315	307	408	336	394
	5	4	301	255	323	377	373	316	201	212	202	360	214	339
	6 (most)	5	546	356	459	444	596	422	246	241	240	460	251	401
		total	2038	2038	2038	2038	2038	2038	2038	2038	2038	2038	2038	2038
Wilcoxon sign rank test		Test statistic	1295218	1025407	1269060.5	1288304.5	1467624.5	1172943.5	671955.5	688419	638317	1293608	734287	1160279
		p-value	1.02E-22	6.07E-01	1.42E-18	1.60E-21	2.51E-60	3.02E-07	8.43E-45	5.70E-41	3.45E-53	2.24E-22	2.48E-31	3.52E-06
		Effect size	0.217	0.011	0.195	0.211	0.363	0.113	0.311	0.297	0.340	0.216	0.258	0.103
		Magnitude	small	small	small	small	moderate	small	moderate	small	moderate	small	small	small
		Sign	+	-	+	+	+	+	-	-	-	+	-	+
Kruskal–Wallis test		Covariate	Effect size											
		Age	small	small	small	NS	NS	small	small	small	small	small	small	small
		Region	NS	NS	NS	NS	NS	NS	small	small	small	NS	small	NS
		Race	NS	NS	NS	NS	NS	NS	small	small	small	NS	small	NS
		Sex/Gender	small	small	NS	NS	NS	small	small	NS	NS	NS	small	small
		Education	small	small	small	small	small	small	small	small	small	small	small	small
		Income	small	small	small	small	small	small	small	small	small	small	small	small
		Political philosophy	small	small	NS	NS	small	NS	NS	NS	NS	small	NS	NS
		DNA experience	small	small	small	small	small	small	small	small	small	small	small	small
		Imaging experience	small	small	small	small	small	small	small	small	small	small	small	small

NS = Not statistically significant.

Effect size *r* for Wilcox sign rank test: small = 0.10 < 0.29; Moderate = 0.30 < 0.49; Large = > 0.49

Effect size e^2 for Kruskal–Wallis test: Small = 0.01 < 0.079; Moderate = 0.08 < 0.259; Large = > 0.259

Sign for Wicoxon Test: + indicates a bias in the distribution toward more comfort; - indicates a bias in the distribution toward less comfort

Table VI. Perceived Trust in Diverse Social Actors to Use Facial Images and Data Responsibly

			Advertisers	Tech Companies	Law Enforcement Agencies	Intelligence Agencies	Health Researchers/ Scientists	Healthcare Providers/ Clinicians	Employers	Schools/ Universities	Retailers	State Government	Federal Government	Foreign Government
Sample count	Trust level	Coding	N											
	Not at all	0	1463	979	734	841	497	476	758	732	1357	951	1064	1878
	Not too much	1	1063	1137	841	895	808	770	1139	1072	1149	1081	972	786
	Somewhat	2	780	1241	1429	1324	1602	1709	1357	1472	838	1202	1137	653
	A great deal	3	570	530	890	815	974	945	605	597	516	642	715	515
		total	3876	3887	3894	3875	3881	3900	3859	3873	3860	3876	3888	3832
Wilcoxon sign rank test		Test statistic	2349360	3136659.5	4366054	3953352	4995415	5133710	3616904	3788893	2341756	3350044	3343820	1827700
		p-value	1.05E-96	1.79E-21	2.31E-17	3.18E-03	1.02E-74	3.09E-86	1.07E-01	5.70E-01	1.05E-94	1.46E-09	1.18E-10	2.51E-172
		Effect size	0.335	0.153	0.136	0.047	0.294	0.315	0.026	0.009	0.332	0.097	0.103	0.452
		Magnitude	moderate	small	small	small	small	moderate	small	small	moderate	small	small	moderate
		Sign	-	-	+	+	+	+	-	+	-	-	-	-
Kruskal–Wallis test		Covariate	Effect size											
		Age	moderate	small	small	small	small	small	small	small	small	small	small	moderate
		Region	small	small	NS	small	small	NS	small	small	small	small	small	small
		Race	small	small	small	NS	NS	NS	small	small	small	small	small	small
		Sex/Gender	small	small	NS	NS	small	small	small	small	small	NS	NS	NS
		Education	small	small	small	small	small	small	small	small	small	small	small	small
		Income	small	small	small	small	small	small	small	small	small	small	small	small
		Political philosophy	small	small	small	NS	small	small	small	small	small	small	small	small
		DNA experience	small	small	small	small	small	small	small	small	small	small	small	small
		Imaging experience	small	small	small	small	small	small	small	small	small	small	small	small

NS = Not statistically significant.
Effect size *r* for Wilcox sign rank test: small = 0.10 < 0.29; Moderate = 0.30 < 0.49; Large = > 0.49
Effect size e^2 for Kruskal–Wallis test: Small = 0.01 < 0.079; Moderate = 0.08 < 0.259; Large = > 0.259
Sign for Wicoxon Test: + indicates a bias in the distribution toward more trust; - indicates a bias in the distribution toward less trust

Post-hoc tests looking at the relationshio between age and trust in advertisers

group1	group2	n1	n2	statistic	p	p.adj	p.adj.signif
18 to 25	26 to 35	490	692	-0.56250	5.74E-01	1.00E+00	ns
	36 to 45	490	653	0.55641	5.78E-01	1.00E+00	ns
	46 to 55	490	647	-3.12365	1.79E-03	3.75E-02	*
	56 to 65	490	651	-9.41018	4.95E-21	1.04E-19	****
	66 to 75	490	622	-12.75682	2.86E-37	6.00E-36	****
	76 +	490	121	-7.29763	2.93E-13	6.15E-12	****
26 to 35	36 to 45	692	653	1.21829	2.23E-01	1.00E+00	ns
	46 to 55	692	647	-2.81335	4.90E-03	1.03E-01	ns
	56 to 65	692	651	-9.69934	3.03E-22	6.37E-21	****
	66 to 75	692	622	-13.34500	1.27E-40	2.66E-39	****
	76 +	692	121	-7.18113	6.91E-13	1.45E-11	****
36 to 45	46 to 55	653	647	-3.97184	7.13E-05	1.50E-03	**
	56 to 65	653	651	-10.76200	5.20E-27	1.09E-25	****
	66 to 75	653	622	-14.34658	1.12E-46	2.35E-45	****
	76 +	653	121	-7.82098	5.24E-15	1.10E-13	****
46 to 55	56 to 65	647	651	-6.76837	1.30E-11	2.74E-10	****
	66 to 75	647	622	-10.39076	2.73E-25	5.74E-24	****
	76 +	647	121	-5.59089	2.26E-08	4.74E-07	****
56 to 65	66 to 75	651	622	-3.70528	2.11E-04	4.43E-03	**
	76 +	651	121	-1.79823	7.21E-02	1.00E+00	ns
66 to 75	76 +	622	121	0.29925	7.65E-01	1.00E+00	ns

Multiple comparison: Dunn test with Bonferroni adjust
NS = Not statistically significant.

Post-hoc tests looking at the relationshio between age and trust in foreign governments

group1	group2	n1	n2	statistic	p	p.adj	p.adj.signif
18 to 25	26 to 35	481	676	1.45896	1.45E-01	1.00E+00	ns
	36 to 45	481	646	1.47485	1.40E-01	1.00E+00	ns
	46 to 55	481	641	-3.81443	1.36E-04	2.87E-03	**
	56 to 65	481	645	-9.47268	2.73E-21	5.73E-20	****
	66 to 75	481	618	-13.14006	1.94E-39	4.08E-38	****
	76 +	481	125	-7.04724	1.83E-12	3.83E-11	****
26 to 35	36 to 45	676	646	0.03258	9.74E-01	1.00E+00	ns
	46 to 55	676	641	-5.75244	8.80E-09	1.85E-07	****
	56 to 65	676	645	-11.94904	6.57E-33	1.38E-31	****
	66 to 75	676	618	-15.91963	4.63E-57	9.73E-56	****
	76 +	676	125	-8.16062	3.33E-16	7.00E-15	****
36 to 45	46 to 55	646	641	-5.72066	1.06E-08	2.23E-07	****
	56 to 65	646	645	-11.84804	2.20E-32	4.63E-31	****
	66 to 75	646	618	-15.77783	4.42E-56	9.28E-55	****
	76 +	646	125	-8.14956	3.65E-16	7.67E-15	****
46 to 55	56 to 65	641	645	-6.10656	1.02E-09	2.14E-08	****
	66 to 75	641	618	-10.09064	6.08E-24	1.28E-22	****
	76 +	641	125	-4.88259	1.05E-06	2.20E-05	****
56 to 65	66 to 75	645	618	-4.05568	5.00E-05	1.05E-03	**
	76 +	645	125	-1.40010	1.61E-01	1.00E+00	ns
66 to 75	76 +	618	125	0.93264	3.51E-01	1.00E+00	ns

Multiple comparison: Dunn test with Bonferroni adjust
NS = Not statistically significant.

Table VII. Perceived Trust in Diverse Social Actors to Use DNA and DNA Data Responsibly

			Advertisers	Tech Companies	Law Enforcement Agencies	Intelligence Agencies	Health Researchers/ Scientists	Healthcare Providers/ Clinicians	Employers	Schools/ Universities	Retailers	State Government	Federal Government	Foreign Government
Sample count	Trust level	Coding	N											
	Not at all	0	1884	1456	915	1052	674	597	1258	1169	1758	1224	1223	1977
	Not too much	1	816	1033	872	836	727	763	1076	1053	951	964	923	691
	Somewhat	2	624	927	1284	1255	1476	1586	1003	1108	675	1114	1025	673
	A great deal	3	534	443	804	722	1013	962	523	510	462	570	687	494
		total	3858	3859	3875	3865	3890	3908	3860	3840	3846	3872	3858	3835
Wilcoxon sign rank test		Test statistic	1864425	2198053.5	3809658	3463207	4713163	4873648	2596430	2728258	1813376	2854038	2993567	1743722
		p-value	2.14E-171	9.41E-115	4.16E-01	4.98E-05	7.39E-43	3.28E-54	9.48E-64	2.90E-47	9.79E-177	2.48E-40	1.54E-27	1.14E-189
		Effect size	0.449	0.367	0.013	0.065	0.220	0.248	0.271	0.233	0.457	0.214	0.175	0.474
		Magnitude	moderate	moderate	small	small	small	small	small	small	moderate	small	small	moderate
		Sign	-	-	+	-	+	+	-	-	-	-	-	-
Kruskal–Wallis test		Covariate	Effect size											
		Age	moderate	small	small	small	small	small	small	small	moderate	small	small	moderate
		Region	small	small	NS	small	small	NS	small	small	small	small	small	small
		Race	small	small	small	NS	small	NS	small	small	small	small	small	small
		Sex/Gender	small	small	small	small	small	small	small	small	small	NS	NS	small
		Education	small	small	small	small	small	small	small	small	small	small	small	small
		Income	small	small	small	small	small	small	small	small	small	small	small	small
		Political philosophy	small	small	small	small	small	small	small	small	small	small	small	small
		DNA experience	small	small	small	small	small	small	small	small	small	small	small	small
		Imaging experience	small	small	small	small	small	small	small	small	small	small	small	small

NS = Not statistically significant.
Effect size *r* for Wilcox sign rank test: small = 0.10 < 0.29; Moderate = 0.30 < 0.49; Large = > 0.49
Effect size e^2 for Kruskal–Wallis test: Small = 0.01 < 0.079; Moderate = 0.08 < 0.259; Large = > 0.259
Sign for Wicoxon Test: + indicates a bias in the distribution toward more trust; - indicates a bias in the distribution toward less trust

Post-hoc tests looking at the relationshio between age and trust in advertisers

group1	group2	n1	n2	statistic	p	p.adj	p.adj.signif
18 to 25	26 to 35	485	694	-0.37198	7.10E-01	1.00E+00	ns
	36 to 45	485	645	1.39878	1.62E-01	1.00E+00	ns
	46 to 55	485	643	-3.66481	2.48E-04	5.20E-03	**
	56 to 65	485	650	-10.18184	2.39E-24	5.02E-23	****
	66 to 75	485	618	-13.24670	4.71E-40	9.90E-39	****
	76 +	485	123	-6.60234	4.05E-11	8.50E-10	****
26 to 35	36 to 45	694	645	1.93964	5.24E-02	1.00E+00	ns
	46 to 55	694	643	-3.62450	2.90E-04	6.08E-03	**
	56 to 65	694	650	-10.78933	3.87E-27	8.12E-26	****
	66 to 75	694	618	-14.13102	2.45E-45	5.14E-44	****
	76 +	694	123	-6.58811	4.45E-11	9.35E-10	****
36 to 45	46 to 55	645	643	-5.46367	4.66E-08	9.79E-07	****
	56 to 65	645	650	-12.50520	6.99E-36	1.47E-34	****
	66 to 75	645	618	-15.76940	5.05E-56	1.06E-54	****
	76 +	645	123	-7.62897	2.37E-14	4.97E-13	****
46 to 55	56 to 65	643	650	-7.02126	2.20E-12	4.62E-11	****
	66 to 75	643	618	-10.35237	4.08E-25	8.57E-24	****
	76 +	643	123	-4.53321	5.81E-06	1.22E-04	***
56 to 65	66 to 75	650	618	-3.42886	6.06E-04	1.27E-02	*
	76 +	650	123	-0.56548	5.72E-01	1.00E+00	ns
66 to 75	76 +	618	123	1.38801	1.65E-01	1.00E+00	ns

Multiple comparison: Dunn test with Bonferroni adjust
NS = Not statistically significant.

Post-hoc tests looking at the relationshio between age and trust in retailers

group1	group2	n1	n2	statistic	p	p.adj	p.adj.signif
18 to 25	26 to 35	483	685	-0.03711	9.70E-01	1.00E+00	ns
	36 to 45	483	642	1.70125	8.89E-02	1.00E+00	ns
	46 to 55	483	643	-3.18906	1.43E-03	3.00E-02	*
	56 to 65	483	650	-8.34560	7.09E-17	1.49E-15	****
	66 to 75	483	617	-11.08881	1.42E-28	2.99E-27	****
	76 +	483	126	-5.27881	1.30E-07	2.73E-06	****
26 to 35	36 to 45	685	642	1.90558	5.67E-02	1.00E+00	ns
	46 to 55	685	643	-3.45691	5.46E-04	1.15E-02	*
	56 to 65	685	650	-9.11569	7.82E-20	1.64E-18	****
	66 to 75	685	617	-12.09829	1.08E-33	2.26E-32	****
	76 +	685	126	-5.42487	5.80E-08	1.22E-06	****
36 to 45	46 to 55	642	643	-5.27835	1.30E-07	2.74E-06	****
	56 to 65	642	650	-10.85182	1.95E-27	4.11E-26	****
	66 to 75	642	617	-13.76746	4.00E-43	8.40E-42	****
	76 +	642	126	-6.47115	9.73E-11	2.04E-09	****
46 to 55	56 to 65	643	650	-5.56139	2.68E-08	5.62E-07	****
	66 to 75	643	617	-8.54707	1.26E-17	2.65E-16	****
	76 +	643	126	-3.44921	5.62E-04	1.18E-02	*
56 to 65	66 to 75	650	617	-3.06629	2.17E-03	4.55E-02	*
	76 +	650	126	-0.27442	7.84E-01	1.00E+00	ns
66 to 75	76 +	617	126	1.48970	1.36E-01	1.00E+00	ns

Multiple comparison: Dunn test with Bonferroni adjust
NS = Not statistically significant.

Post-hoc tests looking at the relationshio between age and trust in foreign governments

group1	group2	n1	n2	statistic	p	p.adj	p.adj.signif
18 to 25	26 to 35	481	683	0.30502	7.60E-01	1.00E+00	ns
	36 to 45	481	647	1.06414	2.87E-01	1.00E+00	ns
	46 to 55	481	640	-4.31902	1.57E-05	3.29E-04	***
	56 to 65	481	648	-9.89236	4.49E-23	9.44E-22	****
	66 to 75	481	612	-13.59931	4.04E-42	8.49E-41	****
	76 +	481	124	-7.28488	3.22E-13	6.76E-12	****
26 to 35	36 to 45	683	647	0.83685	4.03E-01	1.00E+00	ns
	46 to 55	683	640	-5.06748	4.03E-07	8.47E-06	****
	56 to 65	683	648	-11.18772	4.68E-29	9.83E-28	****
	66 to 75	683	612	-15.21396	2.86E-52	6.00E-51	****
	76 +	683	124	-7.70225	1.34E-14	2.81E-13	****
36 to 45	46 to 55	647	640	-5.82413	5.74E-09	1.21E-07	****
	56 to 65	647	648	-11.86527	1.79E-32	3.77E-31	****
	66 to 75	647	612	-15.83199	1.87E-56	3.93E-55	****
	76 +	647	124	-8.13785	4.02E-16	8.45E-15	****
46 to 55	56 to 65	640	648	-6.00657	1.89E-09	3.98E-08	****
	66 to 75	640	612	-10.04703	9.47E-24	1.99E-22	****
	76 +	640	124	-4.82144	1.43E-06	2.99E-05	****
56 to 65	66 to 75	648	612	-4.13887	3.49E-05	7.33E-04	***
	76 +	648	124	-1.41123	1.58E-01	1.00E+00	ns
66 to 75	76 +	612	124	0.96432	3.35E-01	1.00E+00	ns

Multiple comparison: Dunn test with Bonferroni adjust
NS = Not statistically significant.

Table VIII. Acceptability of a Specific Biometric in Societal Contexts

			Law enforcement agencies assessing potential security threats in public spaces	Companies automatically tracking the attendance of their employees	Advertisers seeing how people respond to public advertising displays	Apartment building landlords tracking who enters or leaves their buildings	Schools tracking the attendance of their students	Election officials verifying voter identification at polling places	Retailers automatically identifying customers in its loyalty or rewards programs	Transportation officials identifying travelers at airports and train stations
Sample count	Rating	Coding	N							
	Unacceptable	0	417	725	1001	797	622	525	877	529
	Not Sure	1	472	536	478	548	499	484	544	516
	Acceptable	2	1061	678	447	597	823	915	506	890
		total	1950	1939	1926	1942	1944	1924	1927	1935
Wilcoxon sign rank test		Test statistic	784609.5	475956	323851.5	416407.5	595029	659257.5	350152	631900
		p-value	5.54E-63	2.10E-01	5.14E-48	8.48E-08	1.24E-07	8.91E-25	1.94E-23	9.40E-22
		Effect size	0.379	0.028	0.332	0.122	0.120	0.234	0.227	0.218
		Magnitude	moderate	NS	moderate	small	small	small	small	small
		Sign	+	-	-	-	+	+	-	+
Kruskal–Wallis test		Covariate	Effect size							
		Age	small	NS	small	small	NS	small	small	small
		Region	NS	NS	small	small	small	NS	small	NS
		Race	small	small	small	small	NS	small	small	small
		Sex/Gender	NS	small	NS	NS	NS	small	NS	small
		Education	NS	small	small	small	NS	small	small	small
		Income	small	small	small	small	small	small	small	small
		Political philosophy	NS	NS	NS	NS	NS	NS	NS	NS
		DNA experience	small	small	small	small	small	small	small	NS
		Imaging experience	NS	small	small	NS	small	NS	NS	NS

NS = Not statistically significant.

Effect size r for Wilcox sign rank test: small = 0.10 < 0.29; Moderate = 0.30 < 0.49; Large = > 0.49

Effect size e^2 for Kruskal–Wallis test: Small = 0.01 < 0.079; Moderate = 0.08 < 0.259; Large = > 0.259

Sign for Wicoxon Test: + indicates a bias in the distribution toward more acceptability; - indicates a bias in the distribution toward less acceptability

Table IX. Perceived Effectiveness of a Specific Biometric

			Accurately identifying people	Accurately assessing sex/gender	Accurately assessing race/ethnicity	Accurately detecting emotions	Accurately diagnosing medical conditions
Sample count	Rating	Coding	N				
	Not effective at all	0	133	350	337	607	652
	Not too effective	1	542	958	960	1221	1183
	Somewhat effective	2	1829	1566	1512	1199	1048
	Very effective	3	1290	823	919	649	756
		total	3794	3697	3728	3676	3639
Wilcoxon sign rank test		Test statistic	6146264	4537428	4718948	3429866	3388806
		p-value	<1.00E-100	6.89E-73	7.66E-87	4.11E-01	2.05E-01
		Effect size	0.637	0.297	0.324	0.014	0.021
		Magnitude	large	small	moderate	small	small
		Sign	+	+	+	+	+
Kruskal–Wallis test		Covariate	Effect size				
		Age	small	small	small	small	small
		Region	small	small	small	small	small
		Race	NS	NS	NS	small	small
		Sex/Gender	small	small	small	small	small
		Education	small	small	small	small	small
		Income	small	small	small	small	small
		Political philosophy	small	NS	small	small	small
		DNA experience	small	small	small	small	small
		Imaging experience	small	small	small	small	small

NS = Not statistically significant.

Effect size r for Wilcox sign rank test: small = 0.10 < 0.29; Moderate = 0.30 < 0.49; Large = > 0.49

Effect size e^2 for Kruskal–Wallis test: Small = 0.01 < 0.079; Moderate = 0.08 < 0.259; Large = > 0.259

Sign for Wicoxon Test: + indicates a bias in the distribution toward greater effectiveness; - indicates a bias in the distribution toward lesser effectiveness
